# Editorial: Deciphering Non-Coding Regulatory Variants: Computational and Functional Validation

**DOI:** 10.3389/fbioe.2021.769614

**Published:** 2021-11-03

**Authors:** Li Chen, Mulin Jun Li

**Affiliations:** ^1^ Department of Biostatistics and Health Data Science, Indiana University School of Medicine, Indianapolis, IN, United States; ^2^ Center for Computational Biology and Bioinformatics, Indiana University School of Medicine, Indianapolis, IN, United States; ^3^ Department of Bioinformatics, School of Basic Medical Sciences, Tianjin Medical University, Tianjin, China

**Keywords:** non-coding variants, regulatory variants, variant annotation, functional prediction, functional validation, non-coding variants, functional validation

## 1 Introduction

Non-coding DNA sequences play an important role in an organism’s genome. Genome-wide association studies (GWASs) have produced tens of thousands of genetic variants that are associated with complex phenotypes from different species, and the majority of these associations fall into the non-coding regions and are suggested to be mediated by context-specific regulatory codes. As large-scale genomic sequencing together with constantly evolving biotechnology revolutionize the functional genomics field, we still face great challenges to accurately predict, interpret, and evaluate the biological functions of non-coding regulatory variants in gene regulation. This research topic aims to discuss recent advances made in understanding the regulatory potentials of non-coding genetic variants/somatic mutations (e.g., affecting transcription, splicing, or epigenetic activities) in various organisms using either computational or experimental methods. Novel approaches, tools, and results for accurate regulatory variant prediction and classification, non-coding variant annotation, deleterious prioritization, and functional assays for regulatory mechanism are recommended. Moreover, the research topic is also expanded to seek recent advances in understanding the regulatory potentials of non-coding RNAs.

## 2 Bioinformatics Methods and Analyses for Identifying Functional Effects of Non-coding Regulatory Variants

To pinpoint the non-coding causal variants, Xu et al. developed a computational framework called “regSNPs-ASB” for identifying regulatory single nucleotide polymorphisms (SNPs) in allele-specific transcription factor binding sites from ATAC-seq data. Specifically, regSNPs-ASB simultaneously infers both the genotype information and open chromatin regions from ATAC-seq mapped reads. Then, regSNPs-ASB defines candidate allele-specific transcription factor binding sites by overlapping TF motif binding sites with heterozygous SNPs and open chromatin region. Next, for each candidate allele-specific transcription factor binding site, regSNPs-ASB adopts a generalized linear model (GLM) to model the relationship between ATAC-seq mapped reads as response variable and covariates, which include SNP genotype, the transposase-cleavage event, and the interaction of SNP genotype and transposase-cleavage event. The GLM model aims to test the interaction term to identify heterozygous SNPs with significant allelic imbalance between reference and alternative alleles in open chromatin region, and the heterozygous SNPs with the significant allelic imbalance will be considered as regulatory SNPs. The authors further applied regSNPs-ASB to human MCF-7 breast cancer cells and human mesenchymal stem cells (MSC) and identified 53 and 125 regulatory SNPs respectively. Validated identified regulatory SNPs can affect the expression of their target genes, and the authors found that most of the target genes of regulatory SNPs in promoter regions and target genes of regulatory SNPs in putative enhancer regions in MCF-7 showed significant allele-specific expression. They also found the identified regulatory SNPs in MCF-7 and MSC are enriched in GTEx eQTLs. Based on these discoveries, the author claims that the regSNPs-ASB can be a useful bioinformatics tool to identify candidate causal SNPs from ATAC- seq data only. To study one of the possible mechanisms for non-coding variants affecting gene regulation, Jin et al. leveraged position weight matrixes of transcription factor (TF) and gkm-SVM algorithm to perform a comprehensive evaluation for the functional effects of SNPs on TF binding affinity because the functional consequence of non-coding variants is believed to disrupt the transcription factor binding sites. By exemplifying the predicted binding affinity changes for 18 selected TFs, the authors revealed that the impact of SNPs on TF binding affinity varies substantially and only around 20% of SNPs within putative TF binding sites could have a significant effect on TF binding.

## 3 Bioinformatics Analyses for Studying the Functional Roles of Non-Coding RNAs

The research topic also expands its collection for research works in the non-coding era, which focus on studying the functions of non-coding RNAs. Morenikeji et al. identified 22 conserved long non-coding RNAs (lncRNAs) potentially regulating gene expression in cytokine storm during COVID-19 and found the lncRNA activated by DNA damage is evolutionarily conserved across multiple species, which may serve as potential as targets for intervening in SARS-CoV-2 pathogenesis. Sun et al. identified differential expressed (DE) lncRNAs in salt-tolerant sweet sorghum line (M-81E) and the salt-sensitive line (Roma) and found DE lncRNAs can potentially function as competitive endogenous RNAs to influence plant responses to salt stress. You et al. applied qualitative transcriptional signature to predict CpG island methylator phenotype of right-sided colon cancer. Wang et al. constructed a lncRNA-miRNA-mRNA network and found lncRNA EPB41L4A-AS1 could function as a regulator in the pathogenesis of Non-small Cell Lung Cancer. Chen et al. deciphered the cooperative interactions among these regulatory factors, which include transcription factors, lncRNAs, and microRNAs in Diabetic Nephropathy Progression. All these discoveries demonstrate the importance of non-coding RNAs in gene regulation and biological processes.

